# Androgenic alopecia is associated with higher dietary inflammatory index and lower antioxidant index scores

**DOI:** 10.3389/fnut.2024.1433962

**Published:** 2024-08-15

**Authors:** Sina Bazmi, Matin Sepehrinia, Hossein Pourmontaseri, Hadi Bazyar, Farhad Vahid, Mojtaba Farjam, Azizallah Dehghan, James R. Hébert, Reza Homayounfar, Negin Shakouri

**Affiliations:** ^1^Student Research Committee, Fasa University of Medical Sciences, Fasa, Iran; ^2^Noncommunicable Diseases Research Center, Fasa University of Medical Sciences, Fasa, Iran; ^3^Department of Public Health, Sirjan School of Medical Sciences, Sirjan, Iran; ^4^Nutrition and Health Research Group, Department of Precision Health, Luxembourg Institute of Health, Strassen, Luxembourg; ^5^Department of Epidemiology and Biostatistics, Arnold School of Public Health, University of South Carolina, Columbia, SC, United States; ^6^South Carolina Statewide Cancer Prevention and Control Program, University of South Carolina, Columbia, SC, United States; ^7^National Nutrition and Food Technology Research Institute (WHO Collaborating Center), Faculty of Nutrition Sciences and Food Technology, Shahid Beheshti University of Medical Sciences, Tehran, Iran

**Keywords:** antioxidants, diet, pattern baldness, hair loss, metabolic syndrome, inflammation

## Abstract

**Background:**

Androgenic alopecia (AGA), the most prevalent hair loss type, causes major psychological distress and reduced quality of life. A definite and safe cure/prevention for this condition is still lacking. The role of oxidative stress and inflammation in AGA pathogenesis prompted us to investigate the association between dietary antioxidant index (DAI) and energy-adjusted dietary inflammatory index (E-DII) with AGA.

**Methods:**

The investigation was designed based on data from 10,138 participants from the Fasa Adult Cohort Study (FACS). DAI and energy-adjusted DII (E-DII) were calculated utilizing a validated 125-item food frequency questionnaire (FFQ). A physician diagnosed AGA. Logistic regression models were utilized to evaluate the association of DAI and E-DII with AGA.

**Results:**

After exclusion, 9,647 participants (44.0% men, mean age: 48.6 ± 9.5 years) consisting of 7,348 participants with AGA entered the analyses. Higher DAI was associated with 10% lower AGA odds, while higher E-DII showed 4% higher AGA odds after adjusting for various confounding variables. However, significant associations were found only among women, and adjusting for metabolic syndrome (MetS) made the E-DII-AGA association insignificant.

**Conclusion:**

Antioxidant-rich diets protect against AGA, while pro-inflammatory diets increase the risk, likely through developing MetS. Patient nutrition is frequently overlooked in clinical practice, yet it plays a crucial role, especially for women genetically predisposed to androgenetic alopecia. Dietary changes, such as reducing pro-inflammatory foods (like trans and saturated fats) and increasing anti-inflammatory options (fruits and vegetables), can help prevent hair loss and mitigate its psychological impacts, ultimately lowering future treatment costs.

## 1 Introduction

Androgenic alopecia (AGA) is the most frequent type of hair loss among women and men (1). The prevalence of AGA differs among various ethnicities and escalates with advancing age. Among them, white men are the most affected, with up to 80% experiencing AGA by the age of 70 (1). AGA is a progressive condition characterized by defined hair loss patterns in genetically susceptible individuals (1). The impact of AGA extends beyond cosmetic concerns and impaired self-image; it is also linked to higher odds of cardiovascular diseases (CVD), prostate cancer, polycystic ovary syndrome (PCOS), insulin resistance, hypothyroidism, psychological disorders, and decreased quality of life (2). The pathogenesis of AGA is primarily driven by increased levels of dihydrotestosterone (DHT) in individuals predisposed to the condition. Elevated DHT levels shorten the anagen phase of hair growth and miniaturize the hair follicles in androgen-sensitive areas, resulting in thinning and depigmentation of the hair (3). Currently, one of the main treatment options for AGA involves the use of 5-alpha reductase inhibitors, which convert testosterone into DHT (4). However, discontinuing the medication leads to a recurrence of hair loss. Additionally, 5-alpha reductase inhibitors are associated with adverse effects such as depressed mood, decreased libido, and sexual dysfunction (4). Consequently, researchers continue to explore new strategies for preventing or curing AGA.

Prior investigations have shown a potential correlation between oxidative stress, inflammation, and AGA, opening up possibilities for future treatment and prevention strategies (5). Oxidative stress arises from an imbalance between antioxidants and reactive oxygen species. Antioxidants can be endogenous, such as superoxide dismutase enzymes (SODs), or exogenous, such as selenium and zinc, which are typically obtained through diet (6). Oxidative stress is known to initiate inflammation in the development of AGA, which ultimately leads to the degeneration of hair follicles (7). The involvement of inflammation in AGA pathogenesis has been proposed (8), and the presence of chronic inflammatory infiltrates, particularly in the hair follicles’ upper third, has been confirmed (9). Additionally, heightened inflammation has been linked to a diminished response to drug treatments (10), while the use of anti-inflammatory agents has demonstrated positive outcomes in managing associated alopecia (8).

Maintaining a suitable diet is crucial for the health of hair, as inadequate or imbalanced nutrition has been linked to hair loss (8, 11). Emerging research indicates that regular consumption of soybeans enriched with antioxidants can potentially reduce the risk of AGA (12). Additionally, following a Mediterranean diet and consuming high levels of vegetables and fruits, abundant in antioxidant nutrients, has demonstrated beneficial effects in preventing AGA (13). Moreover, individuals with AGA often exhibit deficiencies in antioxidant elements such as zinc, selenium, and vitamin E (14). Nevertheless, a recent systematic review reported that nutritional interventions and antioxidant supplements do not have a significant impact on AGA (15). Therefore, there are inconsistencies in the findings, and the studies highlight the need for further studies to cover the existing gap. The dietary antioxidant index (DAI) is a measure that evaluates the overall antioxidant effect of the diet based on the intake of vitamin E, vitamin C, vitamin A, zinc, selenium, and magnesium (16). Diets with a low DAI score have been associated with various health conditions, including cardiovascular disease (17) and non-alcoholic fatty liver disease (18).

On the other hand, studies have shown that diet can modulate inflammation and change cytokine levels (19). Consuming a diet rich in trans fatty acids, saturated fatty acids, and high glycemic index carbohydrates is shown to be linked to increased inflammation in the body (19). To assess the potential inflammatory properties of diet based on energy intake micro-and macro-nutrients, the Dietary Inflammatory Index (DII) is designed (20), which has been validated, and various investigations have revealed a considerable link between this index and different health conditions (21). As energy intake is a major component of DII and changes the inflammatory status significantly, the Energy-Adjusted Dietary Inflammatory Index (E-DII) has been proposed (22).

Considering the role of oxidative stress and inflammation in the development of AGA, as well as the proven ability of diet to influence oxidative stress and inflammatory status of human body, we hypothesize that an antioxidant-rich diet and a diet with reduced inflammatory factors could potentially decrease the risk of AGA. Therefore, this study aims to be the first to investigate the link between the E-DII, DAI, and AGA within a large cohort population.

## 2 Materials and methods

### 2.1 Ethics approval and consent to participate

Every individual involved was informed about the aim and subjective of the research and fulfilled the written informed consent. The present study was confirmed by the Ethics Committee of Fasa University of Medical Sciences (Approval Code: IR.FUMS.REC.1402.095) and following the Helsinki Declaration.

### 2.2 Study design

All the eligible participants of the Fasa Adult Cohort Study (FACS; *n* = 10,138) were enrolled in this cross-sectional study. The FACS is an ongoing longitudinal prospective cohort study on adults (age > 35 years old) in Sheshdeh, Fasa, Iran. The participants were asked to complete comprehensive questionnaires, including anthropometrics, medical history, demographic characteristics, and a 125-item food frequency questionnaire (FFQ). The data collection protocol in FACS is explained elsewhere (23, 24). The participants with pregnancy (*n* = 45), missing data (*n* = 111), and very low (< 800 kcal/day: *n* = 33) or very high (> 5,500 kcal/day: *n* = 302) energy intake were excluded from this study.

### 2.3 Measurements

The age (year), gender (male, female), occupational status (employed, unemployed), marital status (single, married, widow, divorced), socioeconomic status (Assert index), body mass index (BMI; kg/m^2^), educational status (illiterate,primary school, secondary school, high school, university), physical activity (metabolic equivalent of tasks, MET), and disease (cardiovascular disease, diabetes mellitus, chronic kidney disease, stroke, myocardial infarction, hypertension, nonalcoholic fatty liver disease), were included from FACS. The Androgenic Alopecia was evaluated by an expert specialist who participated in FACS data gathering.

The metabolic syndrome (MetS) was evaluated using the Adult Treatment Panel III criteria (25), which is suitable for the screening of MetS in large populations due to its high sensitivity. According to this definition, individuals meeting ≥3 of the specified criteria are classified as having MetS: 1. Increased waist circumference (>102 cm in men, >88 cm in women); 2. Increased triglycerides (>150 mg/dL); 3. Low HDL-C (<40 mg/dL in men and < 50 mg/dL in women); 4. High blood pressure (systolic >130 mmHg or diastolic >85 mmHg); 5. Elevated fasting blood sugar (>100 mg/dL).

The participant’s dietary intake was assessed based on a valid semi-quantitative FFQ with 125 food parameters. The FFQ was designed based on the Willett format questionnaire to obtain the dietary intake of participants over a one-year period. The FFQ was modified for Iranian participants according to the common foods consumed by Iranians (23).

### 2.4 Calculation of dietary antioxidant index

The DAI consists of six dietary antioxidants, including vitamins A, E, and C, and minerals zinc, selenium, and manganese, which were calculated based on the FFQ asked from participants of FACS. Each component’s global mean was subtracted from each participant’s individual intake. Then, the calculated figure was divided by the global standard deviation. Eventually, the score of each of the six parameters was summed together to achieve the DAI score of the participants (26).


$$\mathrm{Dietary}\;\mathrm{Antioxidand}\;\mathrm{intake}=\overset{n=6}{\underset{i=1}{\sum }}\frac{\mathrm{individual}\;\mathrm{intake}-\mathrm{global}\;\mathrm{mean}}{\mathrm{gobal}\;\mathrm{standard}\;\mathrm{deviation}}$$

### 2.5 Calculation of energy-adjusted dietary inflammatory index

The consumption value of each nutrient for participants was extracted using recorded FFQ. A regional world database has been provided to obtain the estimated mean and standard deviation (SD) for each parameter of the 34-item E-DII including Vitamin B12, Vitamin B6, Alcohol, Beta Carotene, Cholesterol, Carbohydrate, Caffeine, Energy, n-6 Fatty acids, n-3 Fatty acids, Niacin, MUFA, Mg, Fe, Folic acid, Fiber, Total fat, Garlic, Onions, Proteins, PUFA, Riboflavin, Saturated fat, Se, Thiamin, Trans fat, Vitamin E, Vitamin D, Vitamin C, Vitamin A, Zn, Green/black tea, and peppers. The consumption of pro-and anti-inflammatory factors was calculated using global standards, and the score of each parameter was achieved by multiplying its value by the related coefficient. Finally, achieved scores were deducted from the global standard mean and divided by its SD. Then, E-DII score was obtained using the summation of every item (27).

### 2.6 Statistical analysis

The SPSS v.23 (Armonk, NY: IBM Corp.) was utilized to analyze the present investigation’s data. The numerical data were presented as mean ± standard deviation (SD), and the categorical data were presented as frequency (percent). Since the sample size of present study was large, the distribution of population was considered normal. The chi-square test and independent t-test were utilized to compare the covariates between the two studied groups, the AGA group and the non-androgenic Alopecia group. The covariates with the considerable difference between the two studied groups (*p* < 0.20) were considered as possible covariates. Then, selected covariates were included in the Wald Logistic Regression Model to find the most important covariates for AGA. Eventually, the Enter Logistic Regression Model was applied to assess the link between E-DII and DAI with AGA (with and without considering the final selected covariates). The results were reported as odds ratio (OR) and 95% confidence interval (95% CI). The threshold for statistical significance was set at a *p*-value less than 0.05.

## 3 Results

**Figure 1:**
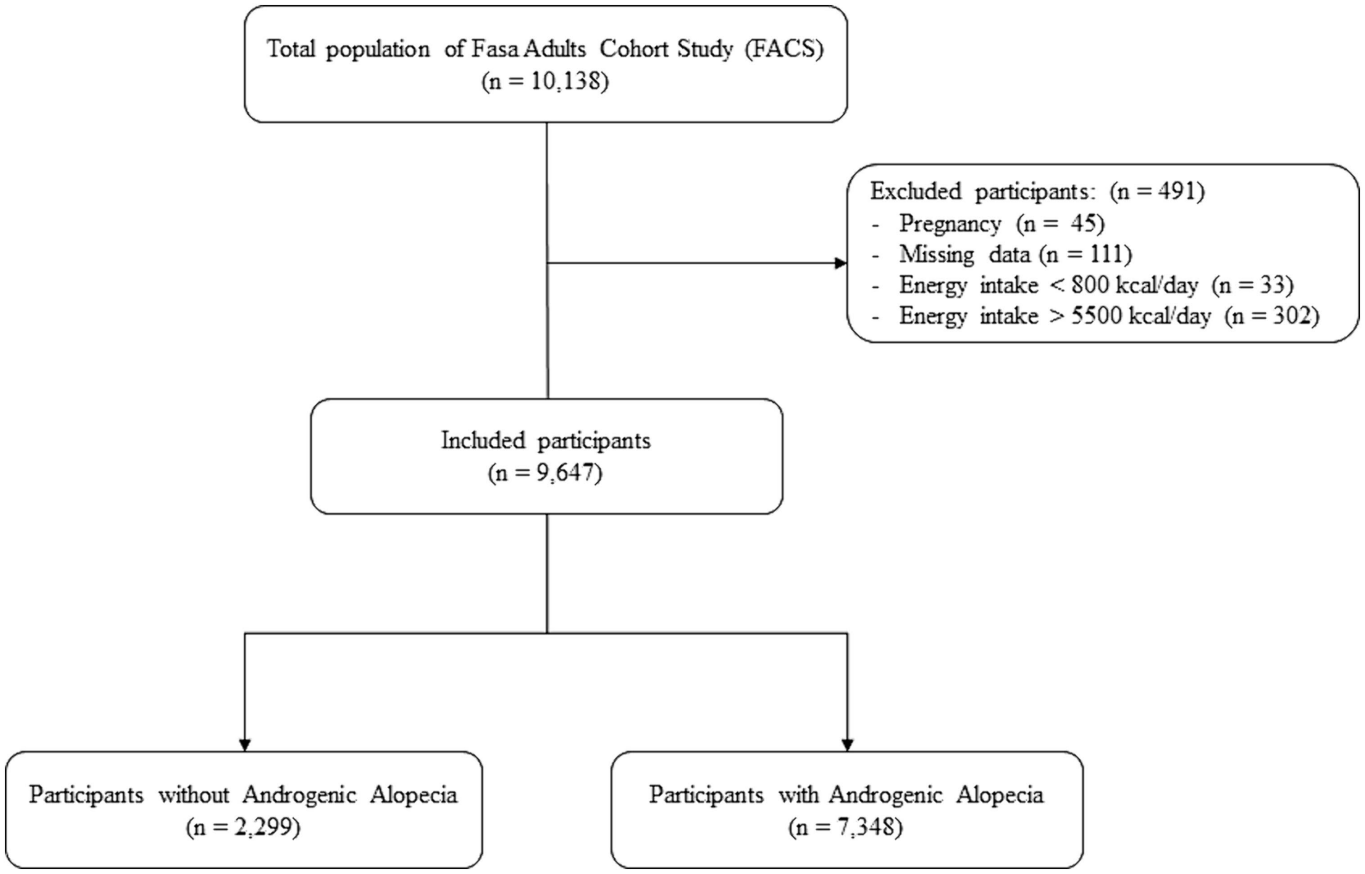
Flow chart of the study population.

The mean age of the final population (*n* = 9,647) was 48.6 ± 9.5 years, comprising of 4,241 (44.0%) men. The mean DAI and E-DII score were 3.52 ± 0.88 and − 0.29 ± 2.06, respectively. Most of the participants were in the AGA group (*n* = 7,348, 76.2%; Figure 1). All the baseline characteristics were significantly different among the two studied groups, except for stroke and chronic kidney disease. The participants with higher age, lower energy intake, worse educational status, different chronic diseases, and no history of smoking or alcohol consumption had a higher frequency of AGA (Table 1).

**Table 1:** Characteristics of the studied population considering androgenic alopecia (*n* = 9,647).

Variable	Subgroup	Total	Non-Androgenic alopecia (*n* = 2,299)	Androgenic alopecia (*n* = 7,348)	*p-*value*
Age (year)		48.6 ± 9.5	46.2 ± 9.2	49.4 ± 9.6	<0.001
Gender	Male	4,241 (44.0)	1,312 (57.1)	2,929 (39.9)	<0.001
	Female	5,406 (56)	987 (42.9)	4,419 (60.1)	
Body mass index (kg/m^2^)		25.7 ± 4.8	25.0 ± 4.8	25 ± 4.8	<0.001
Energy intake (kcal/day)		2,831 ± 937	2,997 ± 975	2,779 ± 918	<0.001
Education	Illiterate	4,406 (45.7)	876 (38.1)	3,530 (48.0)	<0.001
	Primary school	4,445 (46.1)	1,195 (52.0)	3,250 (44.2)	
	Secondary school	631 (6.5)	175 (7.6)	456 (6.2)	
	University	165 (1.7)	53 (2.3)	112 (1.5)	
Smoking (yes)		1820 (18.9)	661 (28.8)	1,159 (15.8)	<0.001
Opium (yes)		1957 (20.3)	702 (30.5)	1,255 (17.1)	<0.001
Alcohol consumption		182 (1.9)	70 (3.0)	112 (1.5)	<0.001
Physical activity (metabolic equivalent of task)		41.3 ± 11.1	42.4 ± 12.8	41.0 ± 10.6	<0.001
Socioeconomic status (Assert index)		0.00 ± 2.11	0.28 ± 2.28	−0.09 ± 2.05	<0.001
Having a Job (yes)		4,878 (50.6)	946 (41.1)	3,932 (53.5)	<0.001
Diabetes (yes)		1,181 (12.2)	212 (9.2)	969 (13.2)	<0.001
Hypertension (yes)		1951 (20.2)	343 (14.9)	1,608 (21.9)	<0.001
Cardiovascular disease (yes)		1,057 (11.0)	184 (8.0)	873 (11.9)	<0.001
Myocardial infarction (yes)		169 (1.8)	29 (1.3)	140 (1.9)	0.040
Stroke (yes)		119 (1.2)	22 (1.0)	97 (1.3)	0.169
Chronic kidney disease (yes)		92 (1.0)	27 (1.2)	65 (0.9)	0.212
Nonalcoholic fatty liver disease (yes)		1,017 (10.5)	195 (8.5)	822 (11.2)	<0.001
Metabolic syndrome (yes)		2,320 (24)	360 (15.7)	1960 (26.7)	<0.001

*The qualitative and quantitative variables were reported as frequency (percent) and mean (standard deviation or standard error). The Independent T-test and Chi-square test were used to compare the covariates between the two studied groups, the Androgenic Alopecia and the non-androgenic Alopecia groups (significant level: *p*-value < 0.05).

Tables 2, 3 indicate that while higher E-DII had a positive significant association with AGA, higher DAI was inversely associated with AGA. After adjusting for BMI, gender, age, educational status, occupational status, socioeconomic status, smoking, opium, and energy intake, both associations between DAI (OR = 0.9; 0.95% CI 0.85, 0.96) and E-DII (OR = 1.04; 0.95% CI 1.01, 1.07) with AGA remained significant. However, after adjusting for MetS, the association between E-DII and AGA became insignificant, though the association between DAI and AGA remained significant. In addition, while all associations were significant among women, the adjusted association between DAI and E-DII with AGA was insignificant among men.

**Table 2:** The association of dietary antioxidant index (continuous and tertiles) and with androgenic alopecia (*n* = 9,647).

Models	Continuous		Tertile				
			T1	T2		T3	
	OR (95%CI)	P		OR (95%CI)	P	OR (95%CI)	P
**Total**							
Model 0	0.88 (0.83, 0.93)	<0.001	1 (ref)	0.79 (0.72, 0.88)	<0.001	0.69 (0.59, 0.82)	<0.001
Model 1	0.90 (0.85, 0.96)	<0.001	1 (ref)	0.80 (0.72, 0.89)	<0.001	0.78 (0.66, 0.92)	0.004
Model 2	0.90 (0.85, 0.96)	<0.001	1 (ref)	0.80 (0.72, 0.89)	<0.001	0.78 (0.66, 0.92)	0.004
**Women**							
Model 0	0.71 (0.65, 0.78)	<0.001	1 (ref)	0.57 (0.49, 0.66)	<0.001	0.50 (0.39, 0.64)	<0.001
Model 1	0.77 (0.70, 0.84)	<0.001	1 (ref)	0.63 (0.53, 0.73)	<0.001	0.59 (0.46, 0.76)	<0.001
Model 2	0.77 (0.70, 0.84)	<0.001	1 (ref)	0.62 (0.53, 0.73)	<0.001	0.60 (0.46, 0.77)	<0.001
**Men**							
Model 0	1.01 (0.94, 1.08)	0.88	1 (ref)	1.00 (0.87, 1.15)	0.977	0.92 (0.74, 1.14)	0.444
Model 1	1.02 (0.94, 1.10)	0.72	1 (ref)	0.99 (0.86, 1.15)	0.923	0.95 (0.75, 1.21)	0.694
Model 2	0.72 (0.94, 1.10)	0.72	1 (ref)	0.99 (0.86, 1.15)	0.942	0.95 (0.75, 1.20)	0.667

Model 0: adjusted for no covariate. Model 1: adjusted for age, gender, body mass index, educational status, occupational status, socioeconomic status, smoking, opium, and energy intake (gender is excluded among women and men analysis). Model 2: adjusted for Model 1 + Metabolic Syndrome. *Logistic regression “enter model” adjusted for covariates with a significant level of *p* < 0.05.

**Table 3:** The association of energy-adjusted dietary inflammatory index (continuous and tertiles) and with androgenic alopecia (*n* = 9,647).

Models	Continuous		Tertile				
			T1	T2	T3		
	OR (95%CI)	P		OR (95%CI)	P	OR (95%CI)	P
**Total**							
Model 0	1.06 (1.04, 1.09)	<0.001	1 (ref)	1.08 (0.97, 1.21)	0.162	1.32 (1.18, 1.48)	<0.001
Model 1	1.04 (1.01, 1.07)	0.020	1 (ref)	1.13 (1.00, 1.28)	0.057	1.18 (1.01, 1.38)	0.037
Model 2	1.03 (0.99, 1.06)	0.190	1 (ref)	1.11 (0.98, 1.26)	0.105	1.11 (0.95, 1.30)	0.177
**Women**							
Model 0	1.03 (1.00, 1.06)	0.052	1 (ref)	1.08 (0.91, 1.28)	0.388	1.13 (0.96, 1.33)	0.131
Model 1	1.09 (1.04, 1.14)	<0.001	1 (ref)	1.24 (1.02, 1.50)	0.028	1.40 (1.12, 1.74)	0.003
Model 2	1.06 (1.01, 1.11)	0.010	1 (ref)	1.16 (0.95, 1.40)	0.142	1.23 (0.98, 1.88)	0.073
**Men**							
Model 0	1.11 (1.07, 1.15)	<0.001	1 (ref)	1.31 (1.12, 1.53)	<0.001	1.57 (1.33, 1.86)	<0.001
Model 1	1.02 (0.97, 1.07)	0.422	1 (ref)	1.12 (0.95, 1.33)	0.188	1.12 (0.91, 1.39)	0.299
Model 2	1.02 (0.97, 1.07)	0.472	1 (ref)	1.12 (0.95, 1.33)	0.178	1.11 (089, 1.37)	0.354

Model 0: adjusted for no covariate. Model 1: adjusted for age, gender, body mass index, educational status, occupational status, socioeconomic status, smoking, opium, and energy intake (gender is excluded among women and men analysis). Model 2: adjusted for Model 1 + Metabolic Syndrome. *Logistic regression “enter model” adjusted for covariates with a significant level of *p* < 0.05.

Additionally, in the final adjusted model, individuals in the last DAI tertile (highest DAI score) had a significantly 22% lower odds of having AGA compared to those in the first tertile of DAI (OR = 0.78; 0.95% CI 0.66, 0.92). This reduction in odds was even more pronounced in women, with 40% lower odds (OR = 0.60; 0.95% CI 0.46, 0.77), but it did not reach statistical significance in men. The adjustment for MetS in the final model did not yield any significant changes in the ORs.

Furthermore, in the second adjusted model, individuals in the last tertile (highest E-DII score) had a significantly 18% higher chance of AGA compared to individuals in the first tertile of E-DII (OR = 1.18; 0.95% CI 1.01, 1.38). This promotion in odds was even stronger in women, with 40% higher odds (OR = 1.40; 0.95% CI 1.12, 1.74), but it was not statistically significant in men. In the final adjusted model, which accounted for MetS, the significance of these associations was also no longer observed in the overall population and among women.

## 4 Discussion

Our results have shown a considerable protective association between the DAI and AGA and a significant adverse association between E-DII and AGA. After subgrouping the results, the associations were completely insignificant among men, while they were significant and more robust among women. After adjusting the associations for the confounding factors, the association between DAI and AGA was still significant. However, adjusting the correlation between E-DII and AGA for MetS made the association insignificant, which reveals that a pro-inflammatory diet likely elevates AGA risk through developing MetS. Therefore, our study indicated that consuming higher amounts of anti-inflammatory and antioxidant-rich foods and micronutrients had a significant impact on preventing AGA, especially among women.

Studies have significantly demonstrated the Mediterranean diet’s association with reduced risk of AGA (11, 28). This diet includes abundant fresh vegetables and fruits, rich in polyphenols with notable antioxidant properties (13). These findings align with results indicating that most patients with AGA report low consumption of fruits and vegetables (8). In contrast, various studies have shown that high doses of supplements commonly containing known micronutrients with antioxidant properties, such as selenium, vitamin E, vitamin C, vitamin A, and zinc (15), can paradoxically stimulate increased hair loss, as demonstrated with vitamin A (29), vitamin E (11), and selenium (30). Nevertheless, the individual association of these antioxidant micronutrients with hair loss has not been precisely determined. Studies on zinc have provided contradictory results (11), while no direct link has been found with vitamin C (11). However, considering the natural composition of nutrients and micronutrients in food, measuring the overall intake of antioxidants provides a more logical perspective (31). Furthermore, the DAI is an accurate indicator for assessing the overall consumption of antioxidants in the diet (32), and this index has shown a significant inverse association with various inflammatory conditions, such as gout (33), diabetes (34), fatty liver (18), and different types of cancers (35–38).

In normal conditions, there is a balance between oxidative stress resulting from repeated exposure to oxidizing agents (39) and the antioxidant system in the keratinocyte cells of hair follicles (28). The onset of AGA begins with the disruption of this balance and the dominance of oxidants over antioxidants (40), leading to elevated production of reactive oxygen species (ROS) in the dermal papilla cells, known as oxidative stress (OS) (28). OS contributes to the pathogenesis of AGA through various mechanisms (15). ROS are highly active molecules that can directly damage all cellular components, including lipids, membranes, proteins, and DNA (41). Furthermore, ROS inhibit the transition from the telogen (resting) phase to the anagen (growth) phase and prematurely induce the entry of hair follicles into the catagen (regression) phase, resulting in growth inhibition and increased apoptosis of hair follicles (28). Additionally, ROS causes premature aging of dermal papilla cells (42), leading to growth arrest and mild inflammation (28). Moreover, OS leads to the production of nitric oxide (NO) and interleukin 1 (IL-1), which intensify and prolong inflammation (43). The inflammation leads to increased free radical production and OS (43), continuing the cycle. Ultimately, OS and inflammation disrupt vascular function and reduce the capacity for vasodilation, resulting in decreased blood supply and oxygen and nutrient delivery to hair follicles, further inhibiting entry into the anagen phase (43, 44). Additionally, the most well-known mechanism of AGA starts with excessive activation of 5-alpha reductase and then dihydrotestosterone (DHT), which leads to the secretion of transforming growth factor-beta 1 (TGF-B1), which in turn increases the production of ROS (OS) (8, 39), and all the aforementioned processes occur. Various studies have demonstrated a considerable decline in antioxidants and a promotion in oxidants in the plasma of AGA patients (43). All of these factors indicate that consuming more anti-inflammatory and antioxidant-rich substances can fundamentally prevent the occurrence of AGA by altering this balance.

After subgrouping the findings of the investigation, the association of E-DII and DAI with AGA was statistically insignificant among men, while higher dietary intake of antioxidants and anti-inflammatory nutrients significantly decreased the chance of AGA among women. A physiological response to androgens, the male sex hormones primarily cause androgenetic alopecia in men. Additionally, men have considerably higher baseline levels of androgens compared to women. It has been shown that most women with androgenetic alopecia do not exhibit signs of increased androgens, and their androgen levels are within the normal range (45, 46). Consequently, other mechanisms apart from androgen increase, such as oxidative stress and inflammation, may play a more prominent role in women, while in men, the impact of androgens is strong enough that other mechanisms have a lower chance of contributing to the development of the condition. Therefore, inhibiting these mechanisms may likely be less effective in disease prevention for men than women.

Additionally, this study shed light on the mediatory role of MetS in the association of E-DII and AGA. Emerging studies revealed that MetS is linked to higher odds of AGA in both females and males (47). Chronic low-grade inflammation is a common pathway in the pathology of many conditions, as well as MetS (48). Also, previous studies indicated a considerable link between the inflammatory potential of diet and MetS (49). Additionally, insulin resistance, the basis of MetS pathophysiology, increases the androgen production from cholesterol and the transformation of testosterone to DHT (50). Therefore, MetS could mediate the connection between E-DII and AGA.

Our study, like other studies, has strengths and limitations. To the best of our knowledge, this study represents the first investigation into the association between the E-DII and DAI with AGA. The data for our study were obtained from a meticulous and comprehensive cohort study, which included a very large sample size. Additionally, we carefully identified and adjusted for potential confounding factors to make our results more reliable. However, due to the study’s cross-sectional nature, despite the association’s logical trend, we cannot establish a causal relationship between AGA and, DAI and E-DII. Future studies utilizing longitudinal analyses can provide a more precise understanding of this relationship. Furthermore, no studies resembling ours assessed the association of DAI and E-DII with AGA, resulting in a lack of directly analogous research in the current literature. Additionally, the progressive stages of AGA were not assessed in this study.

Patient nutrition is often a neglected aspect of clinical practice. Individuals who are genetically predisposed or have a family history of AGA, particularly women, can benefit from dietary modifications. Specifically, they should consider reducing their intake of pro-inflammatory foods such as trans fatty acids and saturated fatty acids while increasing the consumption of anti-inflammatory foods such as fruits and vegetables to help prevent alopecia. This approach may mitigate appearance-related psychological effects and reduce the high costs associated with other medical and non-medical treatments in the future.

## 5 Conclusion

After accounting for confounding factors, this study identified a considerable link between the inflammatory and antioxidant potential of the diet and AGA. The findings indicate that higher consumption of antioxidant-rich foods and lower consumption of high-inflammatory foods in the diet can play a crucial role in preventing AGA. In addition, this study revealed a mediatory role for MetS in the link between diet’s inflammatory potential and AGA. It is recommended that individuals who are genetically predisposed or have a family history of AGA incorporate more antioxidant-rich foods, including fruits and vegetables, and less food with inflammatory properties, such as trans fatty acids and saturated fatty acids, into their diet. This recommendation is especially beneficial for women. Future studies are warranted to delve deeper into the proposed hypothesis and potential long-term effects of diet on preventing AGA.

## Data availability statement

The raw data supporting the conclusions of this article will be made available by the authors, without undue reservation.

## Ethics statement

The studies involving humans were approved by Fasa University of Medical Sciences. The studies were conducted in accordance with the local legislation and institutional requirements. The participants provided their written informed consent to participate in this study.

## Author contributions

SB: Writing – original draft, Writing – review & editing, Conceptualization, Investigation. MS: Conceptualization, Writing – original draft, Writing – review & editing, Investigation. HP: Conceptualization, Methodology, Writing – original draft, Writing – review & editing, Formal analysis. HB: Validation, Writing – review & editing. FV: Supervision, Validation, Writing – review & editing. MF: Data curation, Supervision, Writing – review & editing. AD: Data curation, Writing – review & editing. JH: Writing – review & editing. RH: Data curation, Supervision, Writing – review & editing. NS: Supervision, Validation, Writing – review & editing.
